# Factors Associated with Postoperative Rehospitalization in Patients with Cervical Disc Herniation

**DOI:** 10.3390/ijerph19031687

**Published:** 2022-02-01

**Authors:** Pei-I Lin, Tai-Hsiang Chen, Hsien-Hui Chung, Tsung-Ming Su, Chen-Chung Ma, Tzu-Chi Ou

**Affiliations:** 1 Department of Nursing, Kaohsiung Chang Gung Memorial Hospital, Kaohsiung 833, Taiwan; ymca109@gmail.com; 2 Administrative Office, Weihope Clinic, Kaohsiung 804, Taiwan; scts1215@yahoo.com.tw; 3 College of Management, Yuan Ze University, Taoyuan 320, Taiwan; 4 Department of Pharmacy and Master Program, College of Pharmacy and Health Care, Tajen University, Pingtung County 907, Taiwan; hsienhuichung@gmail.com; 5 Department of Neurosurgery, Kaohsiung Chang Gung Memorial Hospital, Kaohsiung 833, Taiwan; tsungming4516@gmail.com; 6 College of Medicine, Chang Gung University, Kaohsiung 833, Taiwan; 7 Department of Healthcare Administration, I-Shou University, Kaohsiung 824, Taiwan; 8 Department of Medical Education, New Taipei Municipal TuCheng Hospital (Built and Operated by Chang Gung Medical Foundation), New Taipei 236, Taiwan

**Keywords:** cervical disc herniation, demography, rehospitalization

## Abstract

Cervical disc herniation (CDH) is a prevalent disease because of the poor living habits of and great pressure in modern society. Patients experience hand numbness, neck stiffness, soreness, and weakness due to neck nerve root compression, which leads to a gradual increase of neurosurgery outpatients. Although poor posture by the overuse of computers is possibly the origin of CDH, analysis of related factors causing the rehospitalization for CDH patients after surgery in Taiwan is not commonly reported. Thus, the present study focused on the demographics and surgery-related treatment on the relevance of rehospitalization for CDH patients after surgery. The design of the study was retrospective, and we collected data by medical record review, which was derived from the inpatient surgery data of patients at a medical center in southern Taiwan. The study lasted two years from 1 January 2017 to 31 December 2018, and a total of 248 patients underwent surgery for intervertebral disc protrusion in the neck. The retrospective study adopted narrative statistics, the chi-squared test, and binary logistic regression analysis to identify factors affecting postoperative rehospitalization. Among 248 postoperative patients with intervertebral disc protrusion, 178 underwent cervical fusion surgery, and 32 were rehospitalized after surgery for one-year follow up, accounting for an overall prevalence rate of 12.9%. There were no significant differences in sex, age, occupation, hypertension, anterior cervical discectomy and fusion, artificial disc replacement, hybrid surgery, and postoperative cervical coil use (*p* > 0.05). The results of binary logistic regression analysis showed statistically significant differences in abnormal body mass index (*p* = 0.0187, 95% CI = 1.238–10.499), diabetes (*p* = 0.0137, 95% CI = 1.288–9.224) and cervical vertebral surgery hospital days (*p* = 0.0004, 95% CI = 1.028–1.102), predicting the outcome of rehospitalization for CDH patients after surgery. The above results showed that abnormal body mass index, diabetes, and cervical vertebral surgery hospitalization days impacted rehospitalization in CDH patients after surgery. Thus, to prevent diabetes, weight control must be monitored, and maintaining correct posture can reduce CDH and decrease the rate of rehospitalization after surgery, which provides a critical reference for hospital managers and clinical staff.

## 1. Introduction

Many people experience neck pain, stiff shoulders and neck movements, extended shoulder and neck pain or pain in the arm, and even paresthesia and numbness in the arm, which is a disease of modern civilization. The number of patients in neurosurgery clinics is gradually increasing because cervical disc herniation (CDH) has become a more prominent neurological problem in the modern world. According to survey statistics in the United States, CDH is a common clinical spine disease and chronic degenerative disease [1]. In Europe, the number of patients with CDH in men and women increases with age, and the 51- to 60-year-old age group is the most commonly diagnosed patient group [2]. In Taiwan, analysis of the related factors causing rehospitalization for CDH patients after surgery in Taiwan is not commonly reported. There is no significant difference in the prevalence between male and female patients with CDH [3]. The Japanese literature indicated that CDH most often occurs between the fifth, sixth, and seventh cervical vertebrae [4]. In Taiwan, overweight or obesity (BMI ≥ 24 kg/m^2^) [5] is the main risk factor for diabetes, cardiovascular diseases, degenerative spine diseases and postoperative complications [6], and the maintenance of normal weight could reduce the incidence of CDH [7]. CDH patients undergoing surgery are usually diagnosed by neck X-rays and magnetic resonance images (MRI) to assess the compression degree of the spinal cord and nerve roots by the herniated cervical intervertebral disc.

Clinical dynamic cervical radiographs are well-known for predicting the severity of disc herniation in patients with cervical disc herniation after surgery, but further evaluation is needed, although the use of dynamic cervical radiographs to predict disc degeneration and the severity of disc herniation is warranted [8]. Anterior cervical discectomy and fusion (ACDF) remains the standard of surgical treatment to help physicians interpret the diagnosis and avoid rehospitalization [9]. Multiple cervical spondylosis and disc herniation can be detected on MRI, and cervical compression can be detected in 59% of patients on MRI [10]. ACDF surgery can eliminate symptoms and prevent recurrent nerve compression through fusion, reducing readmission rates [11]. In terms of treatment for CDH patients, the commonly used surgical method in neurosurgery is to remove the intervertebral disc and bone spurs through the anterior neck with a precise microscope. To ensure that the nerve is not damaged to achieve nerve root decompression [12], the most important considerations for the height of the intervertebral disc are to maintain the movement of the intervertebral joints and to protect the intervertebral discs in the adjacent segments from premature degeneration [13]. There are two options for rebuilding the space after discectomy; namely, ACDF and artificial disc replacement (ADR), which fills up the space left after the discectomy [14]. Because ACDF may limit cervical spine movement, and joints may lose their original mobility for a lifetime, adjacent segment disease may develop after a long time, causing the recurrence of symptoms and requiring reoperation [15]. Compared with cervical fusion surgery, ADR is a suitable choice for CDH patients. Moreover, artificial cervical discs have similar functions to normal cervical discs, and they are reconstructed at the same time. Additionally, hybrid surgery (ACDF combined with ADR) is an intervertebral disc stent implantation, which can treat multilevel cervical disc degeneration and prevent the degeneration of adjacent segments. Studies found that the fusion rate after hybrid surgery is higher, and the incidence of complications is lower [16]. Postoperative collar usage mainly restricts neck activities to increase the probability of bone healing, and also provides patients with an enhanced sense of security [17]. Moreover, the use of collar support after surgery in the United States can reduce the risk of adjacent segment disease [18]. For ACDF in Taiwan, a fixed intervertebral cage must be worn with a collar for more than 12 weeks, but a movable artificial disc does not require wearing a collar and does not impact normal work and rest. Hospital stay after cervical spine surgery is shortened, and recovery is fast. Most CDH patients in Taiwan are hospitalized for about 1–6 days after surgery, which is similar to the average stay of about 1–6 days from other studies [19].

The present study statistically analyzed the database from a southern Taiwan medical center to compare different related treatments for patients with CDH such as ACDF, ADR, and hybrid surgery. In order to provide medical staff with better practical reference for the postoperative care of CDH patients, the specific purposes of this study are to investigate the postoperative demographic and surgical-treatment-related variables of patients with CDH, and to find factors possibly affecting the rehospitalization of postoperative patients through retrospective statistical analysis.

## 2. Materials and Methods

### 2.1. Data Sources and Research Objects

A retrospective research design was adopted for this study. The research case was based on patients who had been diagnosed with cervical disc herniation in a southern Taiwan medical center from 1 January 2017 to 31 December 2018. In total, 248 patients were included in the study, and their medical records were reviewed. On the basis of the data collection method, this study excluded the following conditions: (1) incomplete medical records; (2) diagnosed as sepsis, tumor spinal lesions, tumors, spinal infections, congenital malformations, and chronic systemic diseases; and (3) herniated cervical intervertebral discs due to trauma. This study was approved by the institutional review board of the Kaoshiung Chang Gung Memorial Hospital on 20 November 2019 (approval no. 201901633B0C601).

### 2.2. Research Variables and Statistical Analysis

This study mainly investigated factors related to the rehospitalization of CDH patients after surgery. The independent variables were (1) demographic variables such as gender, age, occupation, body mass index, diabetes, and hypertension, and (2) surgical-treatment-related variables such as surgical methods (ACDF, ADR, hybrid surgery), postoperative cervical collar usage, and hospital stay after cervical spine surgery. Relatively, the dependent variables contain rehospitalization-related factors. The present study also included patients as follows: the definition of diabetes in this study was that the patient had taken antidiabetic drugs more than half a year before the operation, glycated hemoglobin (HbA1c) ≥ 6.5%, fasting blood glucose ≥ 126 mg/dL, 2 h postmeal blood sugar ≥ 200 mg/dL; the definition of hypertension refers to cases where blood pressure exceeded 140/90 mmHg more than half a year before the operation, and the patient has taken antihypertension drugs; the length of hospital stay for cervical spine surgery was defined as the number of days during which the patient was actually hospitalized for cervical spine surgery (calculation method: (discharge date—operation date) + 1), and the dependent variable, rehospitalization, which was defined as whether the patient had requested unplanned reoperation and had been hospitalized within one year after surgery. The choice of surgical type was not limited and was determined according to the patient’s situation. On the basis of the operational definitions of the independent and dependent variables in the aforementioned research framework, we created a medical-record extraction sheet. Each patient used a separate sheet sorted by the date of surgery and used the medical-record number to retrieve the medical-record data for data extraction and review. Then, we recorded research-related variable information and filled the above results into the medical record extraction sheet. In order to ensure the consistency of the collected data, a review of the medical record data extraction was based on Worster and Haines [20]. Scholars recommended random sampling, taking 10% of the patient data (25 samples) and verifying the data with the same collection methods. After initial verification, there were 3 case data registration errors, and the data consistency rate was 88%. The researcher then immediately corrected the incorrect case data. In order to improve the accuracy of the data, a 10% sample was drawn in the same way, and a second verification was performed. After further verification, there were no case login errors, and the data consistency rate was 100%. According to the statistical analysis of the data from this study, narrative statistics were carried out by frequency and percentage, and independent variables such as demographic variables, ACDF, ADR, and hybrid surgery were discussed by chi-squared test. Correlation between independent variables and the dependent variable (rehospitalization) was analyzed with binary logistic regression to find the predictive factors that affected the quality of medical care after cervical disc herniation surgery. This study used IBM SPSS Statistics version 22 (IBM Corp. Released 2013. IBM SPSS Statistics for Windows, Version 22.0. IBM Corp., Armonk, NY, USA) to process the data, and *p* < 0.05 was judged as a statistically significant difference.

## 3. Results

The results of the study showed that, among 248 postoperative cervical disc herniation patients, regarding the demographic variables, 129 (52%) patients were female; there were 143 (57.7%) people aged 51 to 70; in terms of occupation, 137 people (55.2%) were unemployed; in terms of body mass index: ≥18.5~<24 kg/m^2^, 98 people (39.5%); ≥27 kg/m^2^, 74 people (29.8%); 78 people (31.5%) had diabetes; 107 people (43.1%) had hypertension. In terms of surgery-related treatment changes, 178 people (71.8%) received ACDF during cervical spine surgery; 41 people (16.5%) underwent ADR; 29 people (11.7%) underwent hybrid surgery. Postoperative cervical collar usage: 243 people (98%); 5 people (2.0%) did not use cervical collar after surgery. In terms of length of hospital stay for cervical spine surgery, 143 patients (57.7%) were hospitalized from 1 to 6 days, and 105 patients (42.3%) were hospitalized for over 7 days. In Table 1, the chi-squared test showed that gender, BMI, diabetes, hypertension, and length of hospital stay for cervical spine surgery were statistically significantly associated with rehospitalization.

Among 248 postoperative patients with intervertebral disc protrusion, 32 were rehospitalized after surgery for one-year follow up. All the variables were subjected to binary logistic regression analysis, and the results showed that body mass index, diabetes, and hospitalization days for cervical spine surgery had statistically significant differences in the rehospitalization of patients with cervical disc herniation. Regarding predictors of the outcome of rehospitalization of patients with cervical disc herniation after surgery, in terms of the body mass index, when the patient’s BMI ≥ 24~< 27 kg/m^2^, there was a chance of rehospitalization, which was BMI ≥ 18.5~< 3.61 times of patients with 24 kg/m^2^ (*p* = 0.0187, 95% CI: 1.238–10.499); patients with diabetes had a chance of rehospitalization, which was 3.45 times that of patients without diabetes (*p* = 0.0137, 95% CI: 1.288–9.224); in terms of the number of hospitalization days for cervical spine surgery, the odds of rehospitalization with more than 7 days of hospitalization were 5.19 times the number of hospitalization days (1–6 days; *p* = 0.0004, 95% CI: 1.028–1.102). Table 2 shows research variables through the results of binary logistic regression analysis of the postoperative rehospitalization of patients with cervical disc herniation.

## 4. Discussion

The results of this study showed that the gender incidence of CDH was 52% (129 cases) in females and 48% (119 cases) in males, with similar proportions of different genders. However, the study by Kolenkiewicz et al. [2] found that female incidence was as high as 67%, which may have been caused by demographic characteristics, and that the number of women with CDH in Poland (67%) was higher than the number of men (33%). Kim et al. [7] found that the incidence of CDH in South Korea was higher in women than that in men. Wang et al. [21] also found that the incidence of CDH in women was higher than that of men, accounting for more than 60%. The results of foreign scholars are consistent with the results of this study.

The results of age statistics in this study showed that the age of patients with CDH was 56 (22.6%) 30–50 years old, 143 (57.7%) 51–70 years old, and 49 people ≥71 years old (19.8%) among patients with CDH during the entire study period. Scholars such as Kolenkiewicz et al. [2] showed that the most frequently diagnosed patients were in the 51–60 age group. Kim et al. [7] and the above-mentioned studies showed that the number of male and female CDH patients increases with age, which is consistent with the results of this study.

The body mass index (overweight ≥ 24~<27 kg/m^2^) was 3.61 times the normal body weight (*p* = 0.0187, 95% CI: 1.238~10.499) and research by Dario Muzević et al. [22], and other scholars showed that obesity and overweight are predictors of rehospitalization, which is consistent with the results of this study. In terms of diabetes, the rehospitalization of patients with diabetes was 3.45 times that of patients without diabetes (*p* = 0.0137, 95% CI: 1.288–9.224). Some scholars in the United States studied patients with cervical disc herniation who had undergone surgery, and the incidence of diabetes was higher (*p* < 0.001) as one of the influencing factors [23]. The statistical results of literature data are consistent. There was a statistically significant difference in the number of hospitalization days for cervical spine surgery (number of days during hospitalization is increasing) (*p* ≤ 0.05), which was presented as a predictive factor for rehospitalization of patients with intervertebral disc herniation. If the number of hospitalization days is more than 7 days, the chance of rehospitalization was 5.19 times for the chance of hospitalization stay for 1–6 days (*p* = 0.0004, 95% CI: 2.099–12.815). Compared with patients undergoing cervical spine surgery in the United States, there was a significant difference in the length of hospital stay (*p* < 0.001) [24]. ACDF is still the standard of surgical treatment [25]. Analysis of ACDF predicts the incidence of second surgery in adjacent segments. Patients who have undergone 3 or more segments of arthrodesis are more likely to develop adjacent level disease than patients who have received 1 or 2 segments of anterior cervical arthrodesis are [26]. Cervical fusion surgery uses fixed or artificial disc implantation, both of which have no effect on hospitalization.

This research has limitations. This study adopted the medical history review method. Some variables (such as occupation) are limited by the research data and may not show the true status of the patient. The sample of this study was limited to a southern medical center; the results cannot be extrapolated to patients with CDH in other regions, and rehospitalized patients may not return to the original hospital. This study used secondary data. The difference between the variables cannot explain the causal relationship between variables, and it is impossible to actually understand the true feelings of the patient. According to Wong et al. [27], the choice of fixed and artificial intervertebral discs for the second to seventh cervical spine in three-level hybrid surgery is still controversial and has not been fully studied; future studies could further explore it.

## 5. Conclusions

The distribution of gender and age in this research group, and the incidence of women were higher than those of men. The results of the study are that there are more female patients undergoing CDH surgery. This may be because men only seek medical treatment when their condition is more serious, which leads to delays in seeking medical treatment. The results of this study show that the patient’s body mass index, diabetes, and the length of hospital stay for cervical spine surgery impact the rehospitalization of patients with CDH. Therefore, the results of the study can be used for reference by hospital administrators and medical staff. To clinically strengthen health education for patients, it is necessary to cooperate with medical staff in postoperative health education. For example, guidance on weight control is needed to help prevent cervical disc herniation. In terms of the nursing care of patients, healthcare guidance for postoperative care and precautions for diabetic patients can be strengthened, and the importance of regular follow-up visits and inspections by the doctors can be emphasized. This can be extended to outpatient health education to prevent CDH and maintaining correct posture to avoid disc herniation in the hopes of reducing the rate of rehospitalization of patients after surgery and strengthening the importance of cervical spine healthcare for the general public.

## Figures and Tables

**Table 1:** Chi-squared test of demographic and surgical-treatment-related variables for rehospitalized postoperative cervical disc herniation patients (*N* = 248).

Variable		Number	Percentage	*p* Value
Gender	Male	119	(48%)	0.032 *
	Female	129	(52%)	
Age	30–50 years	56	(22.6%)	0.683
	51–70 years	143	(57.7%)	
	71 years	49	(19.8%)	
Profession	None	137	(55.2%)	0.485
	Workers	32	(12.9%)	
	Freelance	29	(11.7%)	
	Services	50	(20.2%)	
Body mass index	<18.5	8	(3.2%)	0.028 *
	≧18.5~<24	98	(39.5%)	
	≧24~<27	68	(27.4%)	
	≧27	74	(29.8%)	
Diabetes	None	170	(68.5%)	<0.001 ***
	Yes	78	(31.5%)	
Hypertension	None	141	(56.9%)	0.002 *
	Yes	107	(43.1%)	
ACDF		178	(71.8%)	0.090
ADR		41	(16.5%)	0.093
Hybrid surgery		29	(11.7%)	0.662
Use of neck collar after surgery	None	5	(2.0%)	0.385
	Yes	243	(98.0%)	
Length of hospital stay for cervical spine surgery	1–6 days	143	(57.7%)	<0.001 ***
	Over 7 days	105	(42.3%)	

Boldface indicates statistically significant values (*p* value * < 0.05, *p* value *** < 0.001.) Note: ACDF = anterior cervical discectomy and fusion. ADR = artificial disc replacement.

**Table 2:** Logistic regression results of postoperative rehospitalization of patients with cervical disc herniation (*N* = 248).

Variables	Odds Ratio	95% CI		*p* Value
		Lower	Upper	
Gender				
Male	(reference)			
Female	1.786	0.742	4.3	0.1959
Age				
30–50	(reference)			
51–70	0.682	0.216	2.155	0.514
>71	0.76	0.198	2.923	0.6901
Profession				
None	(reference)			
Workers	0.544	0.116	2.548	0.4395
Freelance	0.777	0.155	3.885	0.7584
Services	2.634	0.871	7.966	0.0864
BMI (kg/m2)				
<18.5	(reference)			
≧18.5~<24	5.407	0.535	54.608	0.1526
≧24~<27	3.605	1.238	10.499	0.0187 *
≧27	1.336	0.438	4.079	0.6107
Diabetes				
None	(reference)			
Yes	3.447	1.288	9.224	0.0137 *
Hypertension				
None	(reference)			
Yes	1.666	0.635	4.368	0.2994
Surgery-related treatment variables				
ACDF	(reference)			
ADR	0.514	0.106	2.5	0.4096
Hybrid surgery	1.315	0.357	4.846	0.6803
Use of neck collar after surgery				
None	(reference)			
Yes	2.723	0.034	220.361	0.655
Length of hospital stay for cervical spine surgery				
1–6 days	(reference)			
Over 7 days	5.186	2.099	12.815	0.0004 ***

Note: BMI = body mass index. Boldface indicates statistically significant values (*p* value * < 0.05, *p* value *** < 0.001.). *p* value calculated using logistic regression to determine odds ratio and 95% confidence intervals.

## Data Availability

The data presented in this study are available on request from the corresponding author. The data are not publicly available due to privacy restrictions.

## References

[1] Hilibrand W. (2015). Cervical Radiculopathy: Epidemiology, Etiology, Diagnosis, and Treatment. J. Spinal. Disord. Tech..

[2] Kolenkiewicz M., Włodarczyk A., Wojtkiewicz J. (2018). Diagnosis and Incidence of Spondylosis and Cervical Disc Disorders in the University Clinical Hospital in Olsztyn, in Years 2011–2015. Biomed. Res. Int..

[3] (2017). Reference Guidelines for the Certification of Occupational Cervical Intervertebral Disc Herniation in 106 by the Occupational Safety and Health Administration of the Ministry of Labor. https://www.osha.gov.tw/1106/1176/1185/1190/1191/.

[4] Nakashima H., Yukawa Y., Suda K., Yamagata M., Ueta T., Kato F. (2015). Cervical Disc Protrusion Correlates With the Severity of Cervical Disc Degeneration: A Cross-Sectional Study of 1211 Relatively Healthy Volunteers. Spine.

[5] Hwang L.C., Bai C.H., Chen C.J. (2006). Prevalence of obesity and metabolic syndrome in Taiwan. J. Formos. Med. Assoc..

[6] Chen W., Chen J., Xu J., Su C. (2019). Differences in different body mass index and physical fitness performance of employees in a hospital. Physiotherapy.

[7] Sheng B., Feng C., Zhang D., Spitler H., Shi L. (2017). Associations between Obesity and Spinal Diseases: A Medical Expenditure Panel Study Analysis. Int. J. Env. Res. Public Health.

[8] Kim Y.K., Kang D., Lee I., Kim S.Y. (2018). Differences in the Incidence of Symptomatic Cervical and Lumbar Disc Herniation According to Age, Sex and National Health Insurance Eligibility: A Pilot Study on the Disease’s Association with Work. Int. J. Environ. Res. Public Health.

[9] Wu J.C., Liu L., Huang W.C., Chen Y.C., Ko C.C., Wu C.L., Chen T.-J., Cheng H., Su T.P. (2012). The incidence of adjacent segment disease requiring surgery after anterior cervical diskectomy and fusion: Estimation using an 11-year comprehensive nationwide database in Taiwan. Neurosurgery.

[10] Davies B.M., Mowforth O.D., Smith E.K., Kotter M.R. (2018). Degenerative cervical myelopathy. BMJ.

[11] McClelland S., Passias P.G., Errico T.J., Bess R.S., Protopsaltis T.S. (2017). Outpatient Anterior Cervical Discectomy and Fusion: An Analysis of Readmissions from the New Jersey State Ambulatory Services Database. Int. J. Spine Surg..

[12] Burkus J.K., Traynelis V.C., Haid R.W., Mummaneni P.V. (2014). Clinical and radiographic analysis of an artificial cervical disc: 7-year follow-up from the Prestige prospective randomized controlled clinical trial: Clinical article. J. Neurosurg. Spine.

[13] Skovrlj B., Lee D.-H., Caridi J.M., Cho S.K. (2015). Reoperations Following Cervical Disc Replacement. Asian Spine J..

[14] Lee H., Kim U.C., Oh J.K., Kim T., Park S., Ha Y. (2019). Cost-Effectiveness Analysis of Cervical Anterior Fusion and Cervical Artificial Disc Replacement in the Korean Medical System. J. Korean Neurosurg. Soc..

[15] Teton Z.E., Cheaney B., Bayashi J.T., Than K.D. (2020). PEEK interbody devices for multilevel anterior cervical discectomy and fusion: Association with more than 6-foldhigher rates of pseudarthrosis compared to structural allograft. J. Neurosurg. Spine.

[16] Ryu W.H.A., Platt A., Deutsch H. (2020). Hybrid decompression and reconstruction technique for cervical spondylotic myelopathy: Case series and review of the literature. J. Spine Surg..

[17] Camara R., Ajayi O.O., Asgarzadie F. (2016). Are External Cervical Orthoses Necessary after Anterior Cervical Discectomy and Fusion: A Review of the Literature. Cureus.

[18] Caplan I., Sinha S., Schuster J., Piazza M., Glauser G., Osiemo B., McClintock S., Welch W.C., Sharma N., Ozturk A. (2019). The Utility of Cervical Spine Bracing as a Postoperative Adjunct to Single-level Anterior Cervical Spine Surgery. Asian J. Neurosurg..

[19] Song Z., Zhang Z., Hao J., Shen J., Zhou N., Xu S., Ni W., Hu Z. (2016). Microsurgery or open cervical foraminotomy for cervical radiculopathy? A systematic review. Int. Orthop..

[20] Worster A., Haines T. (2004). Advanced statistics: Understanding medical record review (MRR) studies. Acad. Emerg. Med..

[21] Wang X.R., Kwok T.C., Griffith J.F., Yu B.W.M., Leung J.C., Wáng Y.X.J. (2019). Prevalence of cervical spine degenerative changes in elderly population and its weak association with aging, neck pain, and osteoporosis. Ann. Transl. Med..

[22] Muzević D., Splavski B., Boop F.A., Arnautović K.I. (2018). Anterior Cervical Discectomy with Instrumented Allograft Fusion: Lordosis Restoration and Comparison of Functional Outcomes among Patients of Different Age Groups. World Neurosurg..

[23] Narain A.S., Hijji F.Y., Haws B.E., Kudaravalli K.T., Yom K.H., Markowitz J., Singh K. (2018). Impact of body mass index on surgical outcomes, narcotics consumption, and hospital costs following anterior cervical discectomy and fusion. J. Neurosurg. Spine.

[24] Bhashyam N., De la Garza Ramos R., Nakhla J., Nasser R., Jada A., Purvis T.E., Sciubba D.M., Kinon M.D., Yassari R. (2017). Thirty-day readmission and reoperation rates after single-level anterior cervical discectomy and fusion versus those after cervical disc replacement. Neurosurg. Focus..

[25] Buckland A.J., Baker J.F., Roach R.P., Spivak J.M. (2016). Cervical disc replacement—emerging equivalency to anterior cervical discectomy and fusion. Int. Orthop..

[26] Lee J.C., Lee S.H., Peters C., Riew K.D. (2015). Adjacent segment pathology requiring reoperation after anterior cervical arthrodesis: The influence of smoking, sex, and number of operated levels. Spine.

[27] Wong C.E., Hu H.T., Hsieh M.P., Huang K.Y. (2020). Optimization of Three-Level Cervical Hybrid Surgery to Prevent Adjacent Segment Disease: A Finite Element Study. Front. Bioeng. Biotechnol..

